# Clark Tibbitts Award Lecture

**DOI:** 10.1093/geroni/igaa057.3188

**Published:** 2020-12-16

**Authors:** Cynthia Hancock

## Abstract

The Clark Tibbitts Award lecture will feature an address by the 2020 award recipient, Jan Abusharkrah, PhD, FAGHE. AGHE’s Clark Tibbitts Award was established in 1980 and named for an architect of the field of gerontological education. The award is given each year to an individual or organization that has made an outstanding contribution to the advancement of gerontology and geriatrics education.

